# Investigation of the nutritional and functional roles of a microencapsulated blend of botanicals on intestinal health and growth of nursery pigs challenged with F18^+^*Escherichia coli*

**DOI:** 10.1093/jas/skaf047

**Published:** 2025-02-15

**Authors:** Yesid Garavito-Duarte, Andrea Bonetti, Benedetta Tugnoli, Hyunjun Choi, Andrea Piva, Ester Grilli, Sung Woo Kim

**Affiliations:** Department of Animal Science, North Carolina State University, Raleigh, NC, USA; Department of Animal Science, North Carolina State University, Raleigh, NC, USA; Vetagro S.p.A., Reggio Emilia, Italy; Department of Animal Science, North Carolina State University, Raleigh, NC, USA; Vetagro S.p.A., Reggio Emilia, Italy; Dipartimento di Scienze Mediche Veterinarie, Università di Bologna, Ozzano dell’Emilia, Italy; Vetagro S.p.A., Reggio Emilia, Italy; Dipartimento di Scienze Mediche Veterinarie, Università di Bologna, Ozzano dell’Emilia, Italy; Department of Animal Science, North Carolina State University, Raleigh, NC, USA

**Keywords:** F18^+^*Escherichia coli*, intestinal health, microbiota, nursery pigs, phytobiotics

## Abstract

The study aimed to evaluate the effects of increasing levels of a microencapsulated blend of botanicals (MBB) on the intestinal health and growth performance of nursery pigs challenged with F18^+^*E. coli.* Sixty-four nursery pigs (6.8 ± 0.3 kg) were assigned to 4 dietary treatments in a randomized complete block design, with initial body weight and sex as blocks, and fed for 28 d in 3 phases. Treatments were a basal diet fed to pigs without F18^+^*E. coli* challenge (NC) and 3 levels of MBB (0.0%, 0.1%, and 0.2%) in pigs challenged with F18^+^*E. coli.* On day 7 of the study, pigs in the challenged group were orally inoculated with F18^+^*E. coli* (1.5 × 10^10^ CFU). On days 7 and 21 post-challenge, pigs were euthanized to collect jejunal tissues and mucosa. Compared to the NC, 0.0% MBB increased (*P* < 0.05) relative abundance (RA) of *Staphylococcus saprophyticus* and reduced (*P* < 0.05) *Streptococcus parasuis* at days 7 and 21 post-challenge, respectively. Increasing levels of MBB decreased (linear: *P* < 0.05) RA of *S. saprophyticus* on day 7 post-challenge. Compared to the NC, 0.0% MBB increased (*P* < 0.05) jejunal *NOD2* and IL-6 expression and decreased (*P* < 0.05) *ZO-1* on day 7 post-challenge. Compared to the NC, 0.0% MBB decreased (*P* < 0.05) jejunal IL-6, IL-8, and TNF-α and increased (*P* < 0.05) IgG on day 21 post-challenge. Increasing levels of MBB increased *OCLN* (linear: *P* < 0.05) and *ZO-1* (linear and quadratic: *P* < 0.05) on day 7 post-challenge and decreased *toll-like receptor 4 (TLR4;* linear and quadratic: *P* < 0.05). Compared to the NC, 0.0% MBB decreased (*P* < 0.05) Ki-67^+^ on day 7 post-challenge. Increasing levels of MBB increased (linear: *P* < 0.05) Ki-67^+^ on day 7 post-challenge and villus height (VH):CD on d 21 post-challenge. In the overall period, compared to the NC, 0.0% MBB decreased (*P* < 0.05) average daily gain. Increasing daily MBB intake linearly increased *OCLN* on day 7 and VH:CD on day 21, and reduced *TLR4* and IL-8 on day 21 post-challenge, but exhibiting quadratic effects (*P* < 0.05) on *ZO-1* (optimal at 0.12% of MBB), IgG (optimal at 0.14% of MBB), and G:F during days 7 to 20 and days 7 to 28 (optimal at 0.22% and 0.10% of MBB, respectively). In conclusion, F18^+^*E. coli* challenge negatively modulated the jejunal mucosal microbiota and reduced intestinal morphology and growth of nursery pigs. Supplementation of MBB at 0.10% to 0.14% provided optimal mitigation of the impacts of F18^+^*E. coli* challenge on humoral immunity, intestinal integrity, jejunal morphology, and feed efficiency of pigs.

## Teaser

An oral challenge with F18^+^*E. coli* negatively impacted the intestinal health and growth of pigs, leading to an increased immune response and disrupted intestinal integrity. Increasing levels of a microencapsulated blend of botanicals positively influenced the intestinal health and feed efficiency of nursery pigs, showing a reduction in opportunistic pathogenic bacteria, an enhanced immune response, and improved intestinal integrity and morphology.

## Introduction

In recent years, the pig production industry has shown increased interest in exploring dietary interventions to enhance both the intestinal health and growth performance of nursery pigs (Kim and Duarte, 2021). Postweaning stress can lead to intestinal dysbiosis, increasing susceptibility to enterotoxigenic *Escherichia coli* (*E. coli*) infection. The *E. coli* expressing F18 fimbriae is the primary cause of postweaning diarrhea (PWD) in nursery pigs. This strain produces heat-stable toxins A (STa) and B (STb), which contribute to disease development of pigs (Castro et al., 2022), leading to intestinal inflammation, oxidative stress, and impaired intestinal health and performance (Lugrin et al., 2014; Luppi et al., 2016; Xiong et al., 2019; Kim et al., 2022; Duarte et al., 2023a).

Historically, PWD has been treated with antibiotics and pharmacological doses of zinc oxide to mitigate the symptoms of weaning stress (Rhouma et al., 2017). However, antibiotic administration is currently confined to severe cases and faces restrictions due to public concerns over antimicrobial resistance and the sustainability of food-animal production. This issue establishes a threat to the effectiveness of antibiotics for both human and animal health (Luppi, 2017; Castro et al., 2022). Similarly, the widespread use of high doses of zinc oxide has been prohibited in various regions, including the European Union (Bonetti et al., 2021).

Among innovative approaches, phytobiotics have emerged as potential candidates with multifaceted nutritional and functional roles as antibiotic alternatives (Coddens et al., 2017; Sun and Kim, 2017; Wong et al., 2022). Phytobiotics, a category of botanicals sources, includes essential oils, oleoresins, herbs, and spices, which offer a range of beneficial properties (Kim et al., 2008; Alagawany et al., 2021; Pandey et al., 2023). These compounds have shown the potential to positively affect the intestinal health and growth of nursery pigs during the postweaning period (Li et al., 2021a). Phytobiotics have demonstrated antimicrobial, antioxidant, anti-inflammatory, and immune-modulating properties (Kommera et al., 2006; Chang et al., 2022), with the potential to reduce diarrhea incidence and improve intestinal integrity in nursery pigs (Montoya et al., 2021; Jerez-Bogota et al., 2023).

The bioactive compounds of phytobiotics are susceptible to environmental factors such as light, heat, oxygen, and humidity, which can reduce the properties of phytobiotics (Choi et al., 2019; Christaki et al., 2022). Some phytobiotic compounds possess unpleasant odors and flavors that can reduce appetite (Kommera et al., 2006; Christaki et al., 2022). Phytobiotics administered in their free form can also be quickly absorbed and metabolized in the stomach, limiting the amount that reaches the intestine (Michiels et al., 2008; Anderson et al., 2012; Van Noten et al., 2020), which reduces the beneficial effects of bioactive compounds in phytobiotics on intestinal microbiota and mucosal integrity (Sun and Kim, 2017). Microencapsulation techniques can be employed to mitigate these negative impacts (Abdul Mudalip et al., 2021; Duarte and Kim, 2022). The microencapsulation process embeds bioactive compounds within a protective matrix, providing several technological and biological benefits. For instance, microencapsulation has been shown to protect the bioactive compounds of phytobiotics from degradation during feed processing and storage (Piva et al., 2007; Partheniadis et al., 2017). Additionally, it ensures the targeted release of these bioactive compounds in the small intestine, thereby enhancing their efficacy (Xu et al., 2020; Ambrosio et al., 2022). These properties enhance intestinal integrity and modulate the microbiota by disrupting bacterial membranes (Han and Lee, 2022). Additionally, the bioactive compounds may also reduce oxidative stress (Tsao and Deng, 2004) and inhibit intestinal inflammation (Miguel, 2010), contributing to improved growth performance and health outcomes in pigs (Xu et al., 2020).

Based on previous findings, it was hypothesized that increasing levels of a microencapsulated blend of botanicals (MBB) could mitigate the negative impacts of F18^+^*E. coli* in nursery pigs by positively altering the jejunal mucosa-associated microbiota, enhancing immune response, and reducing oxidative damage, consequently maintaining intestinal morphology and improving growth performance. To test this hypothesis, the objective of this study was to evaluate the effects of increasing levels of MBB on the mucosa-associated microbiota, mucosal immune response, intestinal morphology, diarrhea incidence, and growth performance of nursery pigs challenged with F18^+^*E. coli*.

## Materials and Methods

The experimental protocol was approved by the Institutional Animal Care and Use Committee of North Carolina State University. The blend of botanicals used in this study AviPower5 (Vetagro S.p.A., Reggio Emilia, Italy), is a proprietary blend of selected botanicals microencapsulated in a lipid matrix. The primary bioactive compounds are terpenes and terpenoid molecules, with thymol being the largest constituent by weight.

### Animals, experimental design, and diets

The experiment was conducted at the Metabolism Education Unit at North Carolina State University (Raleigh, NC). Sixty-four nursery pigs (PIC 337 × Camborough 22) at 21 d of age (32 barrows and 32 gilts) with initial body weight (BW) of 6.8 ± 0.3 kg were assigned in a randomized complete block design, with sex and initial BW as blocks. Four dietary treatments (*n* = 16 from days 0 to 14; *n* = 8 from days 14 to 28) were included: a basal diet fed to pigs without F18^+^*E. coli* challenge (NC) and a basal diet with 3 levels of MBB (0.0%, 0.1%, and 0.2%) fed to pigs challenged with F18^+^*E. coli*. The MBB was supplemented by replacing corn in the basal diet. Diets were formulated in 3 phases (phase 1: days 0 to 7; phase 2: days 7 to 20; phase 3: days 20 to 28) and met the nutrient requirements of the NRC (2012), as shown in Table 1. Pigs were individually housed with free access to feed and water for the entire duration of the study. At the end of each phase, nursery pigs and feed disappearance were individually weighed to determine growth performance parameters, including BW, average daily gain (ADG), average daily feed intake (ADFI), and gain-to-feed ratio (G:F). The fecal score was recorded every day during the entire period, based on a 1 to 5 scale: 1) very hard and dry feces, 2) firm stool, 3) normal stool, 4) loose stool, and 5) watery stool with no shape as described by Weaver and Kim (2014). Fecal scores between 1 and 3 represented normal feces, whereas fecal scores of 4 and 5 indicated diarrhea.

**Table 1.** Composition of basal diets (as-fed basis)

Item	Phase 1^1^	Phase 2^2^	Phase 3^3^
Feedstuff, %			
Corn, yellow	43.66	50.11	61.09
Whey permeate	19.00	13.00	6.00
Soybean meal, 48% CP	18.50	22.00	28.50
Poultry meal	9.00	5.00	—
Fish meal	4.00	3.00	—
Blood plasma	3.00	—	—
Enzyme-treated SBM^4^	—	3.00	—
Poultry fat	0.93	1.63	1.40
L-Lys HCl	0.52	0.46	0.46
L-Met	0.24	0.19	0.16
L-Thr	0.17	0.14	0.14
L-Trp	0.02	0.01	—
L-Val	—	—	0.03
Dicalcium phosphate	—	0.38	0.95
Limestone	0.56	0.68	0.87
Vitamin premix^5^	0.03	0.03	0.03
Mineral premix^6^	0.15	0.15	0.15
Salt	0.22	0.22	0.22
Calculated composition			
Dry matter, %	90.69	90.30	89.50
ME, kcal/kg	3,400	3,400	3,350
CP, %	24.40	22.50	19.50
SID Lys, %	1.50	1.35	1.23
SID Met ^+^ Cys, %	0.82	0.74	0.68
SID Trp, %	0.25	0.22	0.20
SID Thr, %	0.88	0.79	0.73
SID Val, %	0.95	0.87	0.78
Ca, %	0.85	0.80	0.70
STTD P, %	0.45	0.40	0.33
Total P, %	0.69	0.65	0.58

^1^days 0 to 7 postweaning.

^2^days 7 to 20 postweaning.

^3^days 20 to 28 postweaning.

^4^Enzyme-treated soybean meal from Hamlet Protein (Findlay, OH, USA).

^5^The vitamin premix provided the following per kilogram of complete diet: 6,613.8 IU of vitamin A as vitamin A acetate, 992.0 IU of vitamin D_3_, 19.8 IU of vitamin E, 2.64 mg of vitamin K as menadione sodium bisulfate, 0.03 mg of vitamin B_12_, 4.63 mg of riboflavin, 18.52 mg of D-pantothenic acid as calcium pantothenate, 24.96 mg of niacin, and 0.07 mg of biotin.

^6^The trace mineral premix provided the following per kilogram of complete diet: 4.0 mg of Mn as manganous oxide, 165 mg of Fe as ferrous sulfate, 165 mg of Zn as zinc sulfate, 16.5 mg of Cu as copper sulfate, 0.30 mg of I as ethylenediamine di-hydroiodide, and 0.30 mg of Se as sodium selenite.

SID, standardized ileal digestible; STTD P, standardized total tract digestible phosphorus.

On days 7 and 8 of the study, 48 nursery pigs (challenged groups: 0.0%, 0.1%, and 0.2% MBB) received 3 separate oral doses of F18^+^*E. coli* (1 mL each), a total of 1.5 × 10^10^ CFU/pig. The dosing schedule was as follows: dose 1 (6.0 × 10^9^ CFU/pig) at 0800 hours on day 7, dose 2 (3.1 × 10^9^ CFU/pig) at 1700 hours on day 7, and dose 3 (5.5 × 10^9^ CFU/pig) at 1700 hours on day 8. Nursery pigs in the unchallenged treatment received a 1 mL dose of sterile saline solution. The inoculated *E. coli* strain was 2,144 (O147: non-motile), originally isolated from pigs with PWD and producing STa and STb toxins (Sun et al., 2021). Cultures of the F18^+^*E. coli* strain were prepared following a standard protocol as previously reported (Duarte et al., 2020; Xu et al., 2022; Jang et al., 2023). To minimize the possibility of cross-contamination, non-challenged pigs were housed in the same room but separated by solid barriers from challenged pigs, and biosecurity protocols, including changing gloves and footwear, were followed between treatment areas. All daily procedures were conducted first with the non-challenged groups before interacting with the challenge group.

### Sample collection

At 2 time points, day 7 post-challenge (day 14 of the study) and day 21 post-challenge (day 28 of the study), 8 pigs per treatment, randomly selected from the 2 blocks within each treatment, were euthanized by captive bolt followed by exsanguination. Jejunal tissues were collected 3 m from the pyloric-duodenal junction, from the jejunum. Two samples of 15 cm of jejunal tissue were rinsed with a sterile 0.9% saline solution. The first jejunal sample was collected by scraping the intestinal mucosa with a glass slide, which was then stored in two 2 mL Eppendorf tubes, immediately placed in liquid nitrogen, and subsequently stored at −80 °C for further analysis of diversity and relative abundance (RA) of jejunal mucosa-associated microbiota and relative gene expression associated with intestinal barrier markers, including *claudin-1* (*CLDN1*), *occludin* (*OCLN*), *zona occludens-1 (ZO-1), nucleotide-binding oligomerization domain containing 1 (NOD1), nucleotide-binding oligomerization domain containing 2 (NOD2), toll-like receptor 2 (TLR2), and toll-like receptor 4 (TLR4).* The second segment was rinsed with 0.9% saline solution and placed in a 50 mL Falcon tube containing 10% buffered formaldehyde to evaluate intestinal morphology.

### Diversity and RA of jejunal mucosa-associated microbiota

The jejunal mucosa samples were sent to Zymo Research Corporation (Irvine, CA, USA) to determine alpha diversity and the RA of mucosa-associated microbiota in the jejunum. Jejunal mucosa samples were used for DNA extraction and 16S rRNA sequencing using the ZymoBIOMICS-96 MagBead DNA kit (Zymo Research). The extracted DNA samples were prepared for targeted sequencing with the Quick-16S Primer Set V3-V4 (Zymo Research) and NGS library Preparation Kit for microbial analysis. These primers were custom-designed by Zymo Research to provide the best coverage of the 16S gene. The final polymerase chain reaction (PCR) products were quantified with quantitative real-time PCR (qPCR) fluorescence readings and pooled together based on equal molarity. The final pooled library was cleaned up with the Select-a-Size DNA Clean & Concentrator (Zymo Research), then quantified with TapeStation (Agilent Technologies, Santa Clara, CA, USA) and Qubit (Thermo Fisher Scientific, Waltham, WA, USA). For sequencing, the final library was sequenced on Illumina NextSeq 2000 (Illumina, San Diego, CA, USA) with a p1 (cat 20075294) reagent kit (600 cycles). The sequencing was performed with a 30% Phix spike-in using the Phix Control kit V3. Unique amplicon sequences were inferred from raw reads using the DADA2 pipeline (Callahan et al., 2016). Chimeric sequences were also removed with the DADA2 pipeline. The depth of sequencing coverage was > 20,000 × sample. Taxonomy was assigned with the Zymo Research Database, a 16S database that is internally designed and curated, as a reference. Alpha diversity (Chao 1, Shannon, and Simpson index) and beta diversity (Bray-curtis distance) were evaluated with MicrobiomeAnalyst (QC, CA) based on Deng et al. (2023). The amplicon sequence variant (ASV) data were transformed to RA for further statistical analysis, and the ASV data with less than 0.50% abundance within each level were combined as “others”.

### Relative mRNA expression of microbial sensing and intestinal integrity in jejunal tissue

Tissue samples from the jejunum (weighing 50 to 100 mg) were homogenized in 1 mL of TRIzol reagent (15-596-026, Invitrogen, Waltham, MA) using the bead mill 24 homogenizer (Thermos Fisher Scientific Inc.). Homogenization involved 2 cycles at 4.5 m/s for 30 s each, with a 20 s interval on ice between cycles, following the method described by Jang et al. (2023). The samples were centrifuged for 10 min at 12,000 × *g* at 4 °C after homogenization. The resulting supernatant was transferred to a 1.5 mL centrifuge tube with 200 µL of chloroform (Thermo fisher Scientific Inc.) and gently vortexed for 1 min. The tubes were then incubated at room temperature for 10 min, followed by centrifugation for 15 min at 12,000 × *g* at 4 °C. A similar procedure was used to preserve the aqueous phase in 200 µL of isopropanol to precipitate RNA, ensuring efficient recovery during subsequent steps. The resulting supernatant was carefully removed, and the tubes were air-dried in a fume hood for approximately 20 minutes until complete evaporation. The yield and quality of the RNA were assessed using spectrometry, as described by Jang et al. (2023). The extracted RNA was reverse transcribed into cDNA using RevertAid First Strand cDNA synthesis (Thermo Fisher Scientific Inc.). qPCR was performed using the CFX connect real-time PCR (RT-qPCR) detection system (BioRad, Hercules, CA, USA), Maxima SYBR Green/ROX qPCR Master Mix (Thermo Fisher Scientific Inc.), and oligonucleotide primers synthesized by Millipore Sigma (Burlington, MA). The thermocycling conditions for RT-qPCR were as follows: an initial incubation at 25 °C for 5 min, followed by 60 min at 42 °C, and a final termination step at 70 °C for 5 min. The primers are listed in Supplementary Table 1. Relative gene expression was normalized using the delta-delta-Ct method, following Jang et al. (2020).

### Immune responses and oxidative damage products in the jejunal mucosa

Mucosa samples collected from the jejunum were weighed (1 g) and placed in 1 mL of phosphate-buffered saline (PBS) on ice. The samples were homogenized using bead mill 24 homogenizer (Thermo Fisher Scientific Inc.) and transferred to new 2 mL microcentrifuge tubes for centrifugation at 14,000 × *g* for 15 min, following the methodology described by Holanda and Kim (2021). After centrifugation, the supernatant was carefully extracted, divided into 5 aliquots, and stored at −80 °C for subsequent analysis.

To proceed with laboratory analysis, total protein content was determined using the Pierce BCA Protein Assay kit (#23225, Thermo Fisher Scientific Inc.) with absorbance readings at 562 nm, following the methodology outlined by Holanda et al. (2020). The resulting protein contents were used for normalization in subsequent colorimetric assays. The malondialdehyde (MDA) content was measured using the OxiSelect TBARS MDA Quantitation assay kit (#STA-330, intra-assay CV of 5% and inter-assay CV of 10%, Cell biolabs Inc.), with absorbance readings at 532 nm, following the protocol described by Moita et al. (2021). The protein carbonyl quantification was performed with the OxiSelect Protein Carbonyl ELISA kit (#STA-310, intra-assay CV of 5% and inter-assay CV of 10%, Cell biolabs, Inc.), with supernatants diluted in PBS to achieve a final concentration of 10 µg protein/mL, as described by Moita et al. (2021), and the absorbance readings were taken at 450 nm, and contents were expressed as nmol/mg protein.

The immunoglobulin G (IgG) and immunoglobulin A (IgA) contents were determined using ELISA kits (E101-104 and E101-102, intra-assay CV of 5% and inter-assay CV of 10%, Bethyl Laboratories, Inc.), with supernatants appropriately diluted to achieve dilution factors of 1:1,600 and 1:400, respectively, as outlined by Holanda et al. (2020). The absorbance readings were taken at 450 nm, with contents reported as µg/mg of protein. The tumor necrosis factor-alpha (TNF-α) content was quantified using the Porcine TNF-α Immunoassay Kit (#PTA00, intra-assay CV of 6.2% and inter-assay CV of 10%, R&D Systems, Minneapolis, MN, USA), following the method described by Cheng et al. (2021). Absorbance readings at 450 nm, corrected at 570 nm, were used to determine the final TNF-α content, expressed as pg/mg protein. The interleukin-6 (IL-6) content was determined with the Porcine IL-6 Duoset ELISA kit (#DY686, intra-assay CV of 7% and inter-assay CV of 10%, R&D systems), and interleukin-8 (IL-8) content with the Porcine IL-8/CXCL8 Duoset ELISA kit (#DY535, CV intra-assay of 7% and CV inter-assay of 10%, R&D Systems), as described by Deng et al. (2023) and Jang and Kim (2019). Prior to analysis, samples were diluted with reagent diluent at 1:5 ratio. The absorbance readings were taken at 450 nm, with correction at 570 nm. All procedures followed the manufacturer’s protocol. Optical density (OD) values were measured using a plate reader (Synergy HT, BioTek Instruments, Winooski, VT) and analyzed with corresponding software (Gen5 Data Analysis Software, BioTek Instruments). Contents for each analyte were calculated by comparing the resulting OD values against the absorbance of standard curves following the provided manual’s guidelines.

### Intestinal morphology and cryptcell proliferation in the jejunum

Jejunal tissues were used for morphological evaluation. Sections extracted from the jejunum of each nursery pig were fixed in a 10% formalin solution for 24 h. Subsequently, the samples were sectioned into 2 longitudinal sections, placed in a cassette, and immersed in a 70% ethanol solution. These sample sections were then shipped to the University of North Carolina School of Medicine Lineberger Comprehensive Cancer Center (Chapel Hill, NC) for further processing, including dehydration, embedding in paraffin, hematoxylin II staining, and immunohistochemistry of Ki-67^+^ proteins, as described by Deng et al. (2023). For sample evaluation, an Olympus CX31 microscope (Lumenera corporation, Ottawa, Canada) and infinity 2-2 digital CCD software were used. For each sample, images capturing 10 intact villi and associated crypts were obtained and measured, as previously detailed (Jang et al., 2020; Cheng et al., 2021). The measurements included villus height (VH) from the top to the junction of the villus and crypt, and crypt depth from the junction of the villus and crypt to the crypt’s bottom. The VH to crypt depth (VH:CD) ratio was determined by dividing the measured VH by crypt depth. The same tissue sections used for intestinal morphology were also used to count the number of Ki-67^+^ cells in the crypt. In the Teledyne Lumenera INFINITY ANALYZE 7 software, the 10 images for each sample were imported, and the number of Ki-67^+^ cells in the crypt was counted. Image analysis was performed by a single person. The averaged results of 10 measurements per nursery pig were reported as a singular value per nursery pig.

### Statistical analysis

A power test was performed to determine the required number of replications needed to achieve statistical significance for an anticipated mean difference of 9% to 10% at *P* < 0.05. This test used a coefficient of variation of 7.5%, based on prior studies involving pigs with a similar genetic background conducted at the same research facility (Duarte et al., 2020; Jang et al., 2023). The power test, assuming a 95% confidence level, indicated that a minimum of 8 replications per treatment was required to achieve 80% power (Aaron and Hays, 2004). Data were analyzed using the MIXED procedure (SAS Inc., Cary, NC, USA). Initial BW and sex were considered as blocks. The statistical model included dietary treatment as a fixed effect and blocks as random effects. The least squares mean of each treatment was calculated. For growth performance post-challenge, BW data from day 7 was used as a covariate. The experimental unit was a pig, individually housed and fed. A preplanned contrast was used to compare the least square means between NC and 0.0% MBB to determine the impact of the F18^+^*E. coli* challenge. Polynomial contrasts were conducted to evaluate the linear and quadratic effects of challenged groups and inclusion levels of MBB (0.0%, 0.1%, and 0.2% MBB). The linear and quadratic effects of daily MBB intake (g/d) on the parameters were evaluated using the RSREG procedure, with significant effects reported when either linear or quadratic values were observed. The optimal level of MBB for the parameters evaluated was calculated in relation to the ADFI. Fecal scores were analyzed using a GLIMMIX procedure including dietary treatment as a fixed effect and blocks as a random effect. Statistical differences were considered significant with *P* < 0.05 and tendencies with 0.05 ≤ *P* < 0.10.

## Results

### Diversity and RA of the jejunal mucosa-associated microbiota

**Figure 1.**
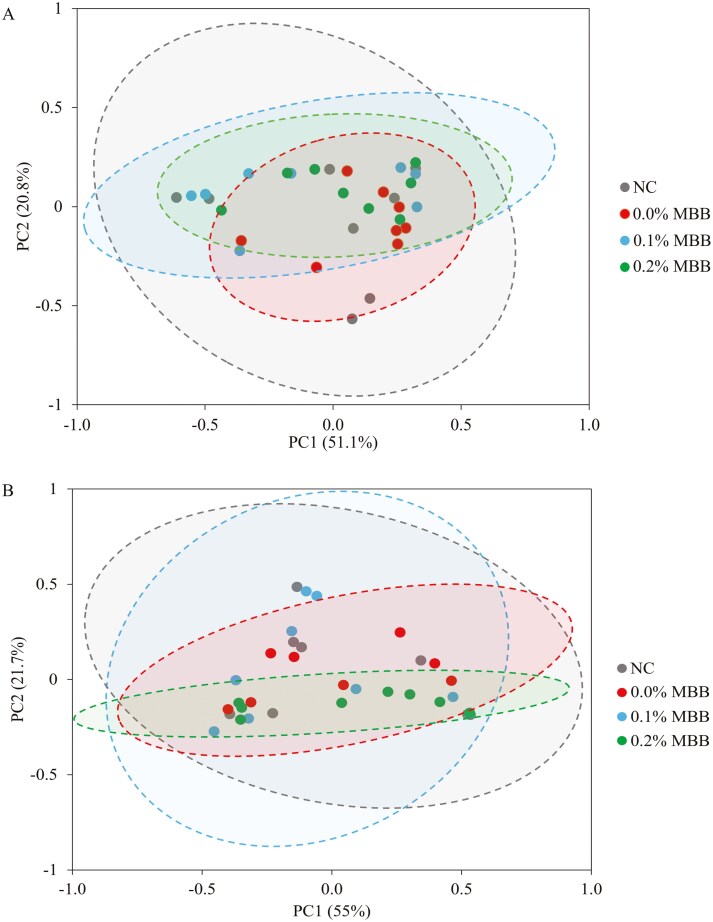
Principal component analysis (PCoA) plot in the jejunal mucosa-associated microbiota at the species level in nursery pigs fed diets supplemented with microencapsulated blends of botanicals (MBB) under F18^+^*E. coli* challenge. The X-axis and Y-axis represent the principal component axes, with the percentages indicating the proportion of variation explained by each component. Points of different colors correspond to samples from different treatments (NC, 0.0%, 0.1%, and 0.2% MBB), and the closer the 2 points are, the more similar their species composition. (**A)** beta diversity in the jejunal mucosa-associated microbiota at the species level in nursery pigs fed diets supplemented with MBB at d 7 post-challenge. The *P* value of the overall test (*P ***= **0.481). The *P* value for Bray-curtis for NC vs. 0.0%: (*P = *0.579), 0.0% vs. 0.1% MBB: (*P *= 0.131), 0.0% vs. 0.2% MBB: (*P *= 0.388); (**B)**: beta diversity in the jejunal mucosa-associated microbiota at the species level in nursery pigs fed diets supplemented with MBB at d 21 post-challenge. The *P* value of the overall test (*P ***= **0.536). The *P* value for Bray-Curtis for NC vs. 0.0%: (*P = *0.592); 0.0% vs. 0.1% MBB: (*P *= 0.609); 0.0% vs. 0.2% MBB: (*P *= 0.370).

There were no differences between the NC and 0.0% MBB for alpha diversity (Table 2) and beta diversity (Figure 1A-B) of mucosa-associated microbiota in the jejunum on days 7 and 21 post-challenge. Increasing levels of MBB tended to have a quadratic effect on the alpha diversity of Chao1 (*P = *0.070), Shannon (*P = *0.055), and Simpson (*P = *0.082) in the jejunal mucosa on day 7 post-challenge. Increasing levels of MBB tended to linearly decrease (*P* = 0.053) the alpha diversity of Shannon in the jejunal mucosa on day 21 post-challenge.

**Table 2.** Alpha diversity of jejunal mucosa-associated microbiota at species level in nursery pigs fed diets supplemented with MBB^1^ under F18^+^*E. coli* challenge

		MBB				*P* value		
Item	NC^2^	0.0%	0.1%	0.2%	SEM^3^	NC vs. 0.0%	Linear^4^	Quadratic^5^
Day 7 post-challenge								
Chao1	237	234	166	313	45	0.962	0.236	0.070
Shannon	4.2	4.9	3.4	4.3	0.5	0.327	0.411	0.055
Simpson	0.8	0.9	0.7	0.8	0.1	0.263	0.366	0.082
Day 21 post-challenge								
Chao1	243	254	218	200	38	0.837	0.276	0.835
Shannon	4.3	5.0	4.8	3.6	0.9	0.369	0.053	0.374
Simpson	0.8	0.9	0.8	0.7	0.1	0.169	0.113	0.968

^1^MBB, microencapsulated blends of botanicals.

^2^NC, basal diet, without F18^+^*E. coli* challenge.

^3^SEM, standard error of means.

^4^Linear, linear effects of increasing levels of MBB under F18^+^*E. coli* challenge.

^5^Quadratic, quadratic effects of increasing levels of MBB under F18^+^*E. coli* challenge.

There were no differences between the NC and 0.0% MBB treatment at the phylum level (Supplementary Table 2) of mucosa-associated microbiota in the jejunum on day 7 post-challenge, whereas, on day 21 post-challenge, compared to the NC, 0.0% MBB treatment tended to increase (*P *= 0.093) RA of Actinobacteria. Increasing levels of MBB tended to have a quadratic effect on the RA of Proteobacteria (*P* = 0.080) and Cyanobacteria (*P* = 0.057) in the jejunal mucosa on day 21 post-challenge.

There were no differences between the NC and 0.0% MBB at the family level (Table 3) of mucosa-associated microbiota in the jejunum on day 7 post-challenge. Increasing levels of MBB linearly decreased (*P *< 0.05) the RA of *Staphylococcaceae* and tended to have a quadratic effect on the RA of *Helicobacteraceae* (*P* = 0.071) in the jejunal mucosa on day 7 post-challenge. On day 21 post-challenge, compared to the NC, 0.0% MBB treatment decreased (*P* < 0.05) the RA of *Streptococcaceae* and tended to increase (*P *= 0.060) the RA of *Bifidobacteriaceae* in the jejunal mucosa. Increasing levels of MBB resulted in a quadratic effect on the RA of *Streptococcaceae* (*P* < 0.05) and *Erysipelotrichaceae* (*P* < 0.05) and tended to have a quadratic effect on the RA of *Ruminococcaceae* (*P *= 0.097) in the jejunal mucosa on day 21 post-challenge.

**Table 3.** Relative abundance of jejunal mucosa-associated microbiota at family level in nursery pigs fed diets supplemented with MBB^1^ under F18^+^*E. coli* challenge

	MBB					*P* value		
Item	NC^2^	0.0%	0.1%	0.2%	SEM^3^	NC vs. 0.0%	Linear^4^	Quadratic^5^
Day 7 post-challenge								
* Helicobacteraceae*	28.3	12.3	41.8	25.7	10.8	0.307	0.335	0.071
* Lactobacillaceae*	22.7	29.7	29.2	36.3	7.8	0.482	0.495	0.647
* Bifidobacteriaceae*	11.8	9.7	11.8	14.5	3.5	0.687	0.303	0.944
* Staphylococcaceae*	10.3	11.1	2.0	0.9	4.7	0.910	0.033	0.310
* Veillonellaceae*	4.3	5.3	3.6	6.2	1.4	0.613	0.627	0.221
* Prevotellaceae*	4.7	4.0	1.6	2.6	1.9	0.760	0.504	0.380
* Coriobacteriaceae*	1.4	1.6	1.2	1.3	0.5	0.755	0.605	0.666
* Lachnospiraceae*	1.9	2.4	1.8	2.3	0.8	0.691	0.935	0.615
* Erysipelotrichaceae*	0.5	1.0	0.7	1.2	0.3	0.172	0.714	0.264
* Leuconostocaceae*	4.6	5.1	0.1	0.2	3.4	0.904	0.318	0.473
* Enterobacteriaceae*	0.2	4.8	0.1	0.5	2.4	0.188	0.280	0.467
* Streptococcaceae*	0.7	0.8	0.3	0.9	0.3	0.772	0.851	0.192
* Ruminococcaceae*	1.0	2.1	0.9	1.4	0.8	0.324	0.557	0.466
* Campylobacteraceae*	0.7	0.9	0.2	0.6	0.6	0.821	0.721	0.400
Others	6.9	9.2	4.7	5.4	2.8	0.573	0.358	0.472
Day 21 post-challenge								
* Helicobacteraceae*	35.2	31.3	19.9	45.0	14.6	0.814	0.381	0.183
* Lactobacillaceae*	18.7	21.0	28.0	30.3	9.0	0.840	0.460	0.820
* Bifidobacteriaceae*	3.6	13.2	9.8	9.4	3.5	0.060	0.494	0.748
* Staphylococcaceae*	7.9	3.4	4.2	1.6	3.9	0.415	0.500	0.449
* Veillonellaceae*	5.6	5.4	6.7	3.0	2.0	0.928	0.413	0.314
* Prevotellaceae*	6.8	7.4	6.6	1.7	3.2	0.899	0.133	0.532
* Coriobacteriaceae*	0.8	1.3	1.8	1.5	0.5	0.572	0.732	0.635
* Lachnospiraceae*	2.5	3.1	3.4	1.4	1.0	0.638	0.149	0.254
* Erysipelotrichaceae*	0.9	1.0	1.8	0.7	0.3	0.744	0.486	0.027
* Streptococcaceae*	5.9	1.1	2.6	0.8	2.2	0.045	0.783	0.039
* Ruminococcaceae*	1.6	1.8	2.7	0.5	0.8	0.797	0.207	0.097
* Peptostreptococcaceae*	2.5	0.5	0.8	0.4	1.3	0.241	0.991	0.497
Others	8.0	9.5	11.7	3.7	3.4	0.715	0.102	0.086

^1^MBB, microencapsulated blends of botanicals.

^2^NC, basal diet, without F18^+^*E. coli* challenge.

^3^SEM, standard error of means.

^4^Linear, linear effects of increasing levels of MBB under F18^+^*E. coli* challenge.

^5^Quadratic, quadratic effects of increasing levels of MBB under F18^+^*E. coli* challenge.

There were no differences between the NC and 0.0% MBB at the genus level (Table 4) of mucosa-associated microbiota in the jejunum on day 7 post-challenge. Increasing levels of MBB tended to have a quadratic effect on the RA of *Helicobacter* (*P* = 0.071) and linearly decreased (*P *< 0.05) the RA of *Staphylococcus* in the jejunal mucosa on day 7 post-challenge. On day 21 post-challenge, compared to the NC, 0.0% MBB treatment tended to increase (*P* = 0.060) the RA of *Bifidobacterium* and decreased (*P* < 0.05) the RA of *Streptococcus* in the jejunal mucosa. Increasing levels of MBB tended to linearly decrease (*P *< 0.05) the RA of *Prevotella* on day 21 post-challenge. Increasing levels of MBB tended to have a quadratic effect on the RA of *Blautia* (*P *=* *0.095) and had a quadratic effect on the RA of *Streptococcus* (*P *< 0.05) in the jejunal mucosa on day 21 post-challenge.

**Table 4.** Relative abundance of jejunal mucosa-associated microbiota at genus level in nursery pigs fed diets supplemented with MBB^1^ under F18^+^*E. coli* challenge

	MBB					*P* value		
Item	NC^2^	0.0%	0.1%	0.2%	SEM^3^	NC vs. 0.0%	Linear^4^	Quadratic^5^
Day 7 post-challenge								
* Lactobacillus*	22.7	29.7	29.2	36.2	7.7	0.482	0.494	0.648
* Bifidobacterium*	11.9	9.7	11.8	14.5	3.5	0.687	0.303	0.303
* Staphylococcus*	10.3	11.2	2.0	0.9	4.7	0.910	0.033	0.310
* Helicobacter*	28.3	12.3	41.6	25.8	10.8	0.307	0.335	0.071
* Megasphaera*	1.1	1.7	1.4	1.5	0.5	0.410	0.784	0.663
* Olsenella*	1.2	1.4	1.1	1.1	0.5	0.803	0.656	0.656
* Weissella*	4.6	5.1	0.1	0.3	3.4	0.904	0.216	0.459
* Mitsuokella*	1.0	1.8	0.9	2.1	0.6	0.300	0.727	0.151
* Streptococcus*	0.7	0.8	0.3	0.9	0.3	0.763	0.869	0.196
* Campylobacter*	0.7	0.9	0.2	0.7	0.6	0.821	0.721	0.400
* Selenomonas*	0.8	0.8	0.5	1.4	0.3	0.948	0.201	0.142
* Escherichia*	0.2	4.8	0.1	0.4	2.4	0.188	0.279	0.467
* Prevotella*	1.0	1.2	0.3	0.5	0.6	0.817	0.316	0.330
Others	15.5	18.6	10.5	13.7	5.2	0.675	0.530	0.404
Day 21 post-challenge								
* Lactobacillus*	18.6	21.1	28.1	30.2	8.9	0.830	0.456	0.818
* Bifidobacterium*	3.6	13.2	9.8	9.4	3.5	0.060	0.494	0.748
* Staphylococcus*	7.7	3.3	4.2	1.6	3.9	0.414	0.500	0.449
* Helicobacter*	35.2	31.3	19.8	45.0	14.6	0.814	0.381	0.183
* Megasphaera*	1.5	1.4	1.8	0.9	0.6	0.907	0.475	0.358
* Olsenella*	0.5	0.9	1.5	1.4	0.5	0.563	0.600	0.607
* Mitsuokella*	1.7	2.3	2.3	1.3	0.8	0.583	0.436	0.590
* Streptococcus*	5.6	1.1	2.6	0.8	2.1	0.039	0.788	0.037
* Dialister*	0.8	0.8	0.8	0.3	0.3	0.977	0.265	0.490
* Romboutsia*	2.5	0.1	0.1	0.4	1.2	0.159	0.309	0.576
* Blautia*	0.7	0.5	0.7	0.1	0.3	0.735	0.136	0.095
* Prevotella*	2.4	3.8	2.7	0.6	1.4	0.465	0.077	0.766
Others	19.2	20.2	25.6	8.0	6.8	0.895	0.077	0.055

^1^MBB, microencapsulated blends of botanicals.

^2^NC, basal diet, without F18^+^*E. coli* challenge.

^3^SEM, standard error of means.

^4^Linear, linear effects of increasing levels of MBB under F18^+^*E. coli* challenge.

^5^Quadratic, quadratic effects of increasing levels of MBB under F18^+^*E. coli* challenge.

Compared to the NC, 0.0% MBB increased (*P* < 0.05) the RA of *Staphylococcus saprophyticus* and tended to increase the RA of *Bifidobacterium dentium* (*P* = 0.053) and *Mitsuokella jalaludini* (*P* = 0.090) of mucosa-associated microbiota in the jejunal mucosa at the species level (Table 5) on day 7 post-challenge. Increasing levels of MBB linearly decreased (*P* < 0.05) the RA of *Staphylococcus saprophyticus* and *Staphylococcus saprophyticus-xylosus*, and tended to linearly increase (*P *= 0.087) the RA of *Lactobacillus mucosae*, whereas tended to linearly decrease (*P *= 0.081) the RA of *Staphylococcus kloosii,* and tended to have a quadratic effect on the RA of *Helicobacter rappini* (*P *= 0.075) in the jejunal mucosa on day 7 post-challenge. On day 21 post-challenge, compared to the NC, 0.0% MBB decreased (*P* < 0.05) the RA of *Streptococcus parasuis* (*P* < 0.05) and tended to increase (*P* = 0.074) the RA of *Bifidobacterium thermacidophilum-thermophilum*. Increasing levels of MBB tended to linearly decrease (*P *= 0.084) the RA of *Prevotella copri* and tended to have a quadratic effect on the RA of *Lactobacillus salivarius* (*P* = 0.096) in the jejunal mucosa on day 21 post-challenge.

**Table 5.** Relative abundance of jejunal mucosa-associated microbiota at species level in nursery pigs fed diets supplemented with MBB^1^ under F18^+^*E. coli* challenge

	MBB					*P* value		
Item	NC^2^	0.0%	0.1%	0.2%	SEM^3^	NC vs. 0.0%	Linear^4^	Quadratic^5^
Day 7 post-challenge								
* Helicobacter rappini*	28.2	12.3	38.1	20.4	10.8	0.304	0.550	0.075
* Bifidobacterium thermacidophilum-thermophilum*	10.0	4.6	7.1	10.4	3.1	0.233	0.123	0.848
* Lactobacillus delbrueckii*	4.4	5.2	9.4	7.5	3.7	0.864	0.645	0.564
* Lactobacillus mucosae*	6.1	6.6	8.9	13.0	2.5	0.929	0.087	0.773
* Staphylococcus kloosii*	3.7	2.2	0.3	0.2	1.5	0.514	0.081	0.313
* Staphylococcus saprophyticus-xylosus*	1.2	1.6	0.5	0.2	0.5	0.630	0.031	0.388
* Bifidobacterium dentium*	<0.1	2.4	0.2	1.5	0.8	0.053	0.507	0.153
* Staphylococcus saprophyticus*	0.6	2.4	0.3	0.1	0.6	0.049	0.025	0.237
* Bifidobacterium boum*	1.8	2.7	4.5	2.1	1.3	0.643	0.790	0.255
* Olsenella profuse*	0.8	1.1	1.0	0.8	0.4	0.620	0.668	0.939
* Mitsuokella multacida*	1.0	0.8	0.5	0.6	0.3	0.641	0.762	0.509
* Mitsuokella jalaludini*	0.1	0.9	0.3	1.1	0.3	0.090	0.656	0.141
* Escherichia coli*	<0.1	4.7	0.1	0.4	2.4	0.170	0.279	0.467
* Weissella thailandensis*	1.5	4.9	1.3	1.5	2.4	0.318	0.220	0.470
* Lactobacillus johnsonii*	1.3	0.4	0.4	1.4	0.5	0.223	0.126	0.363
* Weissella paramesenteroides*	3.0	0.2	<0.1	<0.1	1.5	0.200	0.177	0.238
* Prevotella copri*	0.9	1.0	0.2	0.5	0.5	0.924	0.380	0.338
* Staphylococcus epidermis*	0.7	2.3	0.2	0.1	0.1	0.716	0.194	0.477
* Helicobacter quorum*	<0.1	0.1	3.2	5.2	0.3	1.000	0.878	0.238
Others	34.7	43.7	23.5	33.0	6.5	0.330	0.244	0.089
Day 21 post-challenge								
* Helicobacter rappini*	34.2	29.0	19.9	39.7	14.0	0.751	0.485	0.275
* Bifidobacterium thermacidophilum-thermophilum*	2.3	8.6	7.0	4.7	2.4	0.074	0.323	0.914
* Lactobacillus delbrueckii*	4.6	8.0	16.6	11.8	4.4	0.596	0.583	0.273
* Lactobacillus mucosae*	4.0	5.9	2.7	6.6	2.3	0.566	0.840	0.215
* Staphylococcus kloosii*	5.9	1.4	2.5	1.2	3.1	0.289	0.900	0.290
* Bifidobacterium dentium*	0.1	3.9	0.9	4.2	1.3	0.102	0.504	0.121
* Bifidobacterium boum*	1.3	1.7	1.9	0.6	0.8	0.628	0.312	0.460
* Olsenella profusa*	0.3	0.8	1.0	1.2	0.5	0.465	0.587	0.944
* Helicobacter equorum*	1.1	2.3	0	5.5	1.8	0.642	0.276	0.127
* Mitsuokella multacida*	1.0	0.8	1.0	0.6	0.4	0.654	0.774	0.429
* Lactobacillus salivarius*	1.9	0.6	2.0	0.9	1.0	0.103	0.651	0.096
* Lactobacillus johnsonii*	3.3	0.4	2.4	0.9	1.3	0.136	0.700	0.177
* Streptococcus parasuis*	2.8	0.4	1.1	0.4	0.9	0.023	0.847	0.110
* Rombout siailealis*	2.5	0.1	0.1	0.5	1.2	0.159	0.309	0.576
* Dialister succinatiphilus*	0.8	0.8	0.8	0.3	0.3	0.947	0.266	0.491
* Streptococcus hyointestinalis*	1.7	0.4	0.8	0.4	0.8	0.147	0.861	0.259
* Lactobacillus ruminis*	0.1	1.4	0.5	0.2	0.6	0.114	0.202	0.746
* Prevotella copri*	2.4	3.5	2.4	0.5	1.3	0.517	0.084	0.771
Others	29.7	30.0	36.4	19.8	9.6	0.972	0.239	0.166

^1^MBB, microencapsulated blends of botanicals.

^2^NC, basal diet, without F18^+^*E. coli* challenge.

^3^SEM, standard error of means.

^4^Linear, linear effects of increasing levels of MBB under F18^+^*E. coli* challenge.

^5^Quadratic, quadratic effects of increasing levels of MBB under F18^+^*E. coli* challenge.

### Relative mRNA expression of microbial sensing and intestinal integrity in jejunal tissue

**Figure 2.**
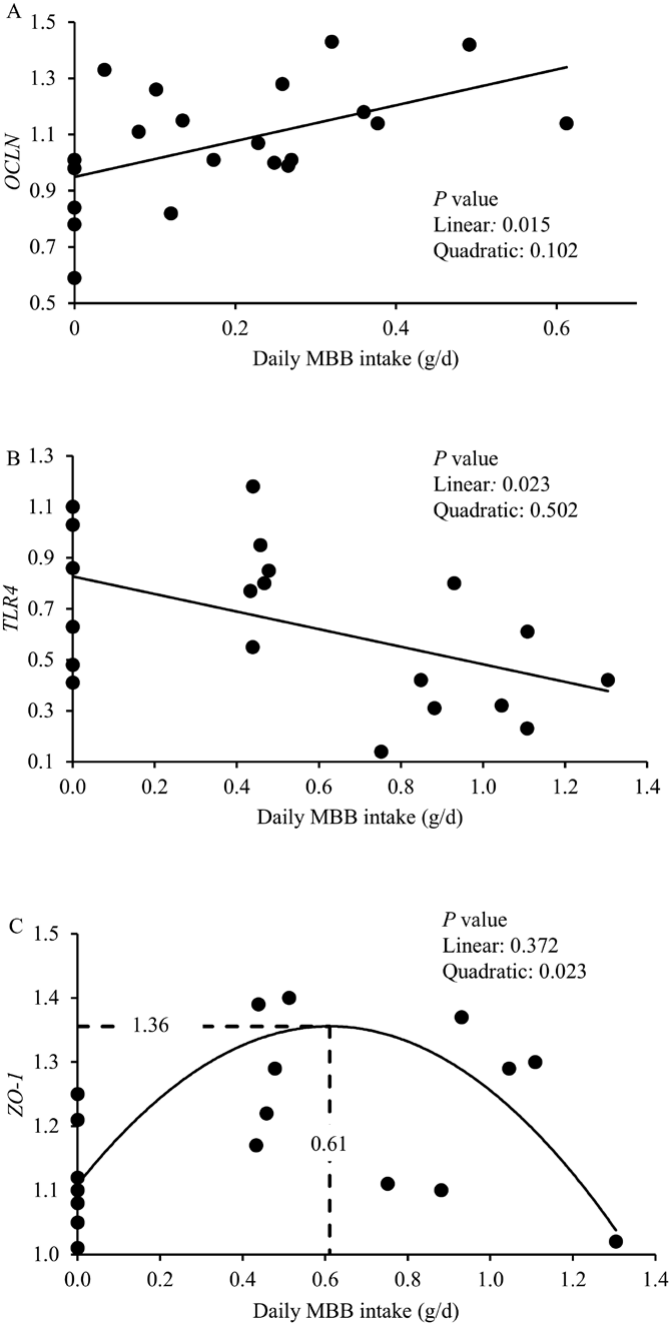
Relative gene expression of intestinal markers of nursery pigs fed with microencapsulated blends of botanicals (MBB) under F18^+^*E. coli* challenge. (**A)** Relative gene expression of *OCLN* of nursery pigs fed diets with increasing levels of MBB intake (g/d) on day 7 post-challenge. Relative gene expression of *OCLN* = 0.95 + 0.66 × MBB intake (g/d). *P* value of MBB intake (g/d): 0.015. The number of observations was 21. (**B)** Relative gene expression of *TLR4* of nursery pigs fed diets with increasing levels of MBB intake, g/d on day 21 post-challenge. *TLR4* gene expression = 0.83 – 0.32 × MBB intake (g/d). *P* value of MBB intake (g/d): 0.023. (**C)** Relative gene expression of *ZO-1* of nursery pigs fed diets with increasing levels of MBB intake (g/d) on day 21 post-challenge. *ZO-1* gene expression = 1.29 × MBB intake (g/d) × MBB intake (g/d)—0.44 × MBB intake (g/d) + 0.65. *P* value of MBB intake (g/d): 0.023. The optimal MBB level for relative gene expression of *ZO-1* in the jejunum of nursery pigs was 0.12% calculated in relation to the average daily feed intake (497 g/d).

On day 7 post-challenge, no differences were observed among the experimental groups for the relative gene expression of *NOD1* and *TLR2* in the jejunal tissue. Compared to the NC, 0.0% MBB decreased (*P* < 0.05) the relative gene expression of *ZO-1* and tended to decrease (*P *= 0.072) the relative gene expression of *OCLN*, whereas it increased (*P* < 0.05) the relative gene expression of *NOD2* on day 7 post-challenge (Table 6). Increasing levels of MBB linearly increased (*P* < 0.05) the relative gene expression of *OCLN* and *ZO-1* and tended to linearly decrease (*P *= 0.064) the relative gene expression of *TLR4*, on day 7 post-challenge. Increasing daily MBB intake (g/d) linearly increased (*P* < 0.05) the relative gene expression of *OCLN* in the jejunal mucosa on day 7 post-challenge (Figure 2A).

**Table 6.** Relative gene expression of microbial sensing and intestinal integrity in the jejunum of nursery pigs fed diets supplemented with MBB^1^ under F18^+^*E. coli* challenge

Item^2^	MBB				SEM^4^	*P* value		
	NC^3^	0.0%	0.1%	0.2%		NC vs. 0.0%	Linear^5^	Quadratic^6^
Day 7 post-challenge								
* NOD1*	1.12	1.12	1.41	1.18	0.17	0.991	0.804	0.223
* NOD2*	1.06	1.37	1.44	1.32	0.16	0.039	0.670	0.454
* TLR2*	1.02	1.01	1.34	1.20	0.18	0.946	0.436	0.275
* TLR4*	1.04	1.27	0.88	0.84	0.14	0.277	0.064	0.388
* CLDN1*	1.02	0.87	1.04	1.04	0.22	0.620	0.583	0.731
* OCLN*	1.00	0.84	1.08	1.21	0.06	0.072	0.001	0.470
* ZO-1*	1.01	0.84	1.12	1.09	0.05	0.047	0.007	0.006
Day 21 post-challenge								
* NOD1*	1.03	0.97	1.28	1.02	0.16	0.768	0.831	0.201
* NOD2*	1.03	1.10	1.09	1.13	0.16	0.772	0.901	0.911
* TLR2*	1.05	0.92	0.87	1.00	0.11	0.411	0.551	0.464
* TLR4*	1.01	0.75	0.88	0.41	0.07	0.034	0.016	0.008
* CLDN1*	1.03	1.02	0.91	1.38	0.22	0.960	0.531	0.577
* OCLN*	1.01	1.25	1.46	1.34	0.08	0.071	0.521	0.148
* ZO-1*	1.01	1.10	1.35	1.20	0.06	0.299	0.298	0.022

^1^MBB, microencapsulated blends of botanicals.

^2^NOD1, nod-like receptor 1; NOD2, nod-like receptor 2; TLR2, toll-like receptor 2; TLR4, toll-like receptor 4; CLDN1, claudin-1; OCLN, occludin; ZO-1, zonula occludens-1.

^3^NC, basal diet, without F18^+^*E. coli* challenge.

^4^SEM, standard error of means.

^5^Linear, linear effects of increasing levels of MBB under F18^+^*E. coli* challenge.

^6^Quadratic, quadratic effects of increasing levels of MBB under F18^+^*E. coli* challenge.

On day 21 post-challenge, no differences were observed among the treatment groups for the relative gene expression of *CLDN1*, *NOD1*, *NOD2*, and *TLR2* in the jejunal tissue. Compared to the NC, 0.0% MBB treatment tended to increase (*P* = 0.071) the relative gene expression of *OCLN* and decreased (*P* < 0.05) the relative gene expression of *TLR4*. Increasing levels of MBB had a quadratic effect on the relative gene expression of *TLR4* (*P* < 0.05) and *ZO-1* (*P* < 0.05) on day 21 post-challenge. Increasing daily MBB intake (g/d) linearly decreased (*P* < 0.05) the relative gene expression of *TLR4* in the jejunal mucosa on day 21 post-challenge (Figure 2B) and had a quadratic effect on the relative gene expression of *ZO-1* (*P* < 0.05) on day 21 post-challenge (Figure 2C). The optimal level of MBB for *ZO-1* in the jejunum was 0.12% calculated in relation to the ADFI (497 g/d).

### Immune responses and oxidative damage products in the jejunal mucosa

**Figure 3.**
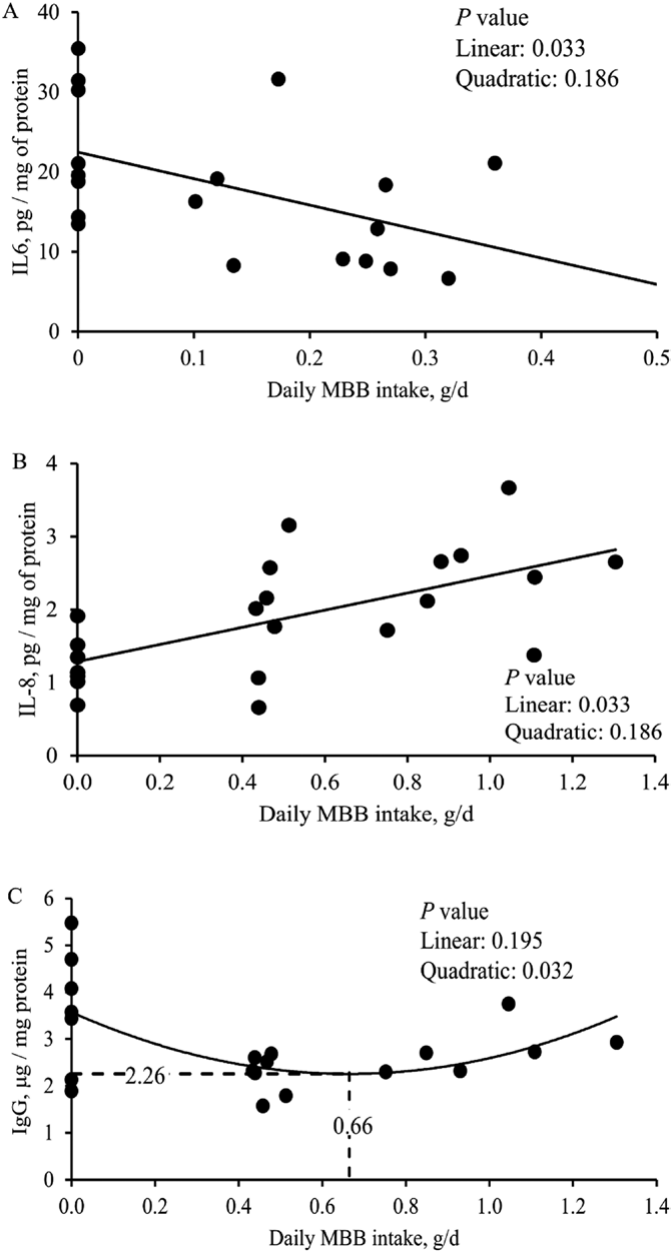
Oxidative stress and immune parameters in the jejunal mucosa of nursery pigs fed with microencapsulated blends of botanicals (MBB) under F18^+^*E. coli* challenge. (**A)** IL-6 levels in the jejunal mucosa of nursery pigs fed with increasing levels of MBB intake, g/d at day 7 post-challenge. IL-6 levels = 22.5 – 33.1 × MBB intake, g/d. *P* value of MBB intake, g/d: 0.033. The number of observations was 19. (**B)** IL-8 levels in the jejunal mucosa of nursery pigs fed diets with increasing levels of MBB intake, g/d at day 21 post-challenge. IL-8 levels = 1.29 + 1.14 × MBB intake, g/d. *P* value of MBB intake, g/d: 0.002. The number of observations was 22. (**C)** IgG levels in the jejunal mucosa of nursery pigs fed diets with increasing levels of MBB intake, g/d at day 21 post-challenge. IgG, μg/mg protein = 2.26 × MBB, g/d × MBB intake, g/d + 2.98 × MBB intake, g/d + 0.67. *P* value of MBB intake, g/d: 0.032. The number of observations was 20. The optimal MBB level for jejunum IgG levels of nursery pigs was 0.14% calculated in relation to the average daily feed intake (497 g/d).

On day 7 post-challenge there were no differences among the experimental groups for IL-8, TNF-α, IgA, IgG, and protein carbonyl contents in the jejunal mucosa. Compared to the NC, 0.0% MBB increased (*P* < 0.05) IL-6 content and tended to increase (*P* = 0.089) MDA content in jejunal mucosa (Table 7) on day 7 post-challenge. Increasing levels of MBB tended to linearly decrease (*P = *0.082) the IL-6 content in jejunal mucosa on day 7 post-challenge. Increasing daily MBB intake (g/d) linearly decreased (*P* < 0.05) the IL-6 content in jejunal mucosa on day 7 post-challenge (Figure 3A).

**Table 7.** Immune responses and oxidative damage products in the jejunal mucosa of nursery pigs fed diets supplemented with MBB^1^ under F18^+^*E. coli* challenge

Item^2^^,^^3^	MBB				SEM^5^	*P* value		
	NC^4^	0.0%	0.1%	0.2%		NC vs. 0.0%	Linear^6^	Quadratic^7^
Day 7 post-challenge (unit/mg protein)								
IL-6, pg	13.87	23.08	15.21	13.83	3.44	0.022	0.082	0.396
IL-8, pg	2.26	1.52	2.06	1.82	0.34	0.119	0.392	0.200
TNF-α, pg	3.95	3.65	3.14	4.47	0.58	0.707	0.321	0.211
IgA, μg	2.95	2.54	3.00	2.26	0.46	0.512	0.673	0.302
IgG, μg	4.94	4.54	3.88	4.44	0.70	0.662	0.913	0.498
MDA, μmol	0.39	0.52	0.48	0.44	0.05	0.089	0.385	0.966
Protein carbonyl, nmol	3.39	3.09	2.92	3.05	0.29	0.345	0.896	0.614
Day 21 post-challenge (unit/mg protein)								
IL-6, pg	19.62	11.71	13.52	13.80	2.50	0.028	0.590	0.804
IL-8, pg	2.50	1.25	2.05	2.42	0.38	0.031	0.004	0.493
TNFα, pg	4.92	2.38	2.54	3.58	0.60	0.002	0.173	0.593
IgA, μg	3.95	6.61	6.06	4.87	1.21	0.073	0.259	0.803
IgG, μg	2.12	3.48	2.37	2.55	0.30	0.003	0.158	0.063
MDA, μmol	0.36	0.35	0.38	0.40	0.04	0.765	0.323	0.915
Protein carbonyl, nmol	2.31	2.41	2.12	2.44	0.34	0.837	0.966	0.449

^1^MBB, microencapsulated blends of botanicals.

^2^IL-6, interleukin-6; IL- 8, interleukin-8; TNF-α, tumor necrosis factor-alpha; IgA, immunoglobulin A; IgG, immunoglobulin G; MDA, malondialdehyde.

^3^NC, basal diet, without F18^+^*E. coli* challenge.

^4^SEM, standard error of means.

^5^Linear, linear effects of increasing levels of MBB under F18^+^*E. coli* challenge.

^6^Quadratic, quadratic effects of increasing levels of MBB under F18^+^*E. coli* challenge.

On day 21 post-challenge there were no differences among the treatments for MDA and protein carbonyl contents in the jejunal mucosa. Compared to the NC, 0.0% MBB decreased (*P* < 0.05) TNF-α, IL-6, and IL-8 contents, increased (*P* < 0.05) IgG content, and tended to increase (*P* = 0.073) IgA content in jejunal mucosa on day 21 post-challenge. Increasing levels of MBB tended to have a quadratic effect on IgG content (*P = *0.064) and linearly increased (*P* < 0.05) the IL-8 content in jejunal mucosa on day 21 post-challenge. Increasing daily MBB intake (g/d) linearly increased (*P* < 0.05) the IL-8 content (Figure 3B) and had a quadratic effect (*P* < 0.05) on IgG content (minimum 2.26 μg/mg of protein at 0.66 g/d MBB intake) in jejunal mucosa on day 21 post-challenge (Figure 3C). The optimal level of MBB for jejunal IgG was 0.14% calculated in relation to the ADFI (497 g/d).

### Intestinal morphology and crypt cell proliferation in the jejunum

On day 7 post-challenge, there were no differences in jejunal VH, crypt depth, and VH:CD among the treatments (Table 8). Compared to the NC, 0.0% MBB decreased (*P* < 0.05) Ki-67^+^ proliferative cell counts in the crypt of jejunum. Increasing levels of MBB linearly increased (*P* < 0.05) the Ki-67^+^ on day 7 post-challenge.

**Table 8.** Intestinal morphology and crypt cell proliferation in nursery pigs fed diets supplemented with MBB^1^ under F18^+^*E. coli* challenge

Item	MBB				SEM^3^	*P* value		
	NC^2^	0.0%	0.1%	0.2%		NC *vs.* 0.0%	Linear^4^	Quadratic^5^
Day 7 post-challenge								
Villus height, µm	417	410	377	413	19.0	0.778	0.904	0.196
Crypt depth, µm	134	142	129	136	4.27	0.209	0.184	0.546
VH:CD^6^	3.12	2.91	2.93	3.03	0.14	0.294	0.568	0.824
Ki-67^+^, unit	72.9	59.2	68.5	68.8	4.0	0.013	0.047	0.270
Day 21 post-challenge								
Villus height, µm	464	412	440	459	33	0.075	0.140	0.876
Crypt depth, µm	157	168	153	152	8.0	0.358	0.184	0.546
VH:CD	2.66	2.50	2.87	3.13	0.23	0.573	0.024	0.763
Ki-67^+^, unit	78.6	79.2	75.5	76.1	2.6	0.861	0.330	0.425

^1^MBB, microencapsulated blends of botanicals.

^2^NC, basal diet, without F18^+^*E. coli* challenge.

^3^SEM, standard error of means.

^4^Linear, linear effects of increasing levels of MBB under F18^+^*E. coli* challenge.

^5^Quadratic, quadratic effects of increasing levels of MBB under F18^+^*E. coli* challenge.

^6^VH:CD, villus height to crypt depth ratio.

**Figure 4.**
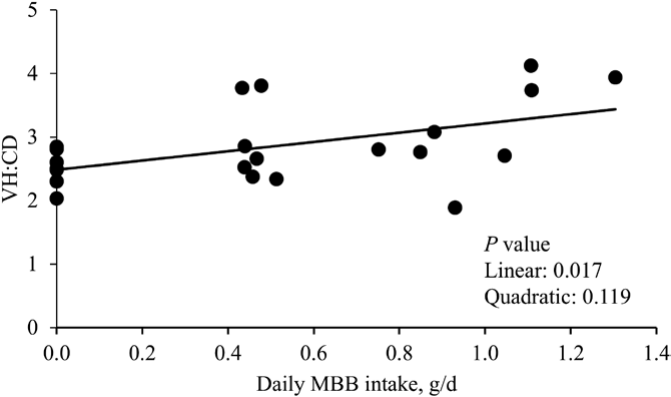
Intestinal morphology of jejunum in nursery pigs fed with microencapsulated blends of botanicals (MBB) under F18^+^*E. coli* challenge. The villus height to crypt depth (VH:CD) ratio in the jejunal mucosa of nursery pigs fed diets with increasing levels of MBB intake, g/d at day 21 post-challenge. VH:CD = 2.49 + 0.73 × MBB intake, g/d. *P* value of MBB intake, g/d: 0.017. The number of observations was 22.

On day 21 post-challenge, compared to the NC, 0.0% MBB treatment tended to decrease (*P* = 0.075) VH (Table 8). There were no differences in crypt depth among the treatments on day 21 post-challenge. Increasing levels of MBB linearly increased (*P* < 0.05) the VH:CD on day 21 post-challenge. Increasing daily MBB intake (g/d) linearly increased (*P* < 0.05) the VH:CD on day 21 post-challenge (Figure 4). There was no difference among treatments for Ki-67^+^ proliferative cell count.

### Fecal score

**Figure 5.**
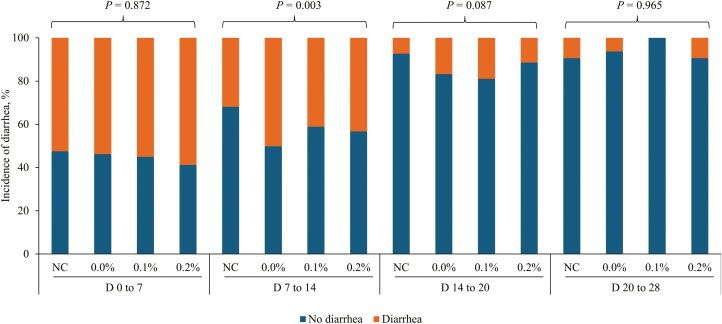
Incidence of diarrhea in nursery pigs fed diets supplemented with microencapsulated blends of botanicals (MBB) from 0% to 0.2% (0, 0.1%, and 0.2%) under F18^+^*E. coli* challenge. Fecal scores were: 1) very hard and dry feces, 2) firm stool, 3) normal stool, 4) loose stool, and 5) watery stool with no shape. Considering fecal score between 1 and 3 as no diarrhea and 4 and 5 as diarrhea.

There were no differences in fecal scores among treatments during the pre-challenge period (Figure 5). During days 7 to 14, differences were observed among treatments (*P* < 0.05) with 0.0% MBB treatment showing a high incidence of diarrhea (50.2%) when compared with 0.1% and 0.2% MBB treatments (41.1% and 43.3%, respectively). From days 14 to 20, the fecal scores were tended to be different (*P* = 0.087), whereas no differences were observed from days 20 to 28.

### Growth performance

**Figure 6.**
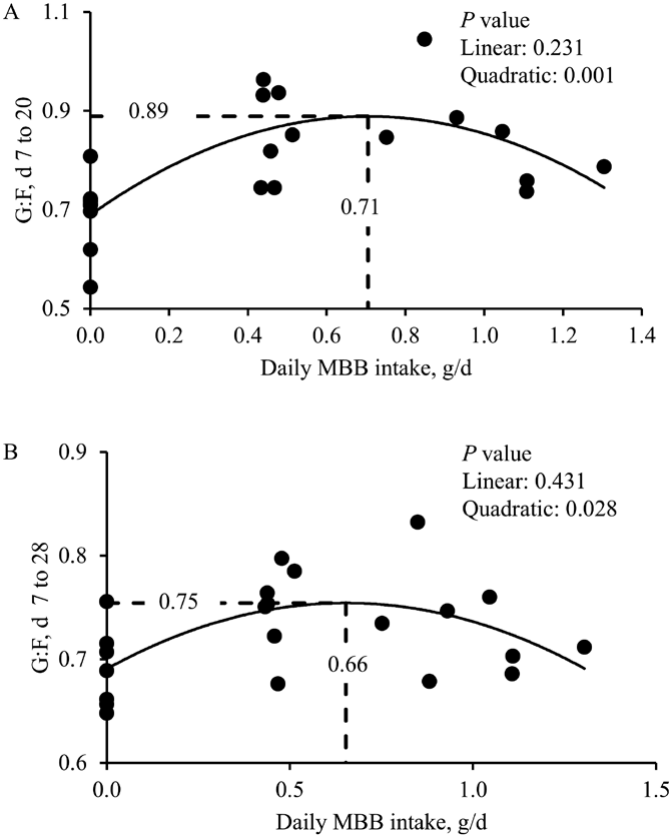
Feed efficiency in nursery pigs fed with microencapsulated blends of botanicals (MBB) under F18^+^*E. coli* challenge. (**A)** G:F ratio of nursery pigs fed diets with increasing levels of MBB intake, g/d in days 7 to 20 of the study. G:F = 0.89 × MBB, g/d × MBB intake, g/d—0.40 × MBB intake, g/d + 0.71. *P* value of MBB intake, g/d: 0.001. The number of observations was 22. The optimal MBB level for G:F of nursery pigs was 0.22% calculated in relation to the average daily feed intake (322 g/d). (**B)** G:F ratio of nursery pigs fed diets with increasing levels of MBB intake, g/d in days 7 to 28 of the study. G:F = 0.75 × MBB, g/d × MBB intake, g/d—0.15 × MBB intake, g/d + 0.65. *P* value of MBB intake, g/d: 0.028. The number of observations was 22. The optimal MBB level for G:F of nursery pigs was 0.10% calculated in relation to the average daily feed intake (622 g/d).

There were no differences among treatments during the pre-challenge period (Table 9). Compared to the NC, 0.0% MBB treatment decreased (*P* < 0.05) BW on day 14 of the study (day 7 post-challenge). During the first week post-challenge (days 7 to 14), compared to the NC, 0.0% MBB treatment decreased (*P* < 0.05) ADG and G:F ratio and tended to decrease (*P *= 0.054) ADFI. Increasing levels of MBB tended to linearly increase (*P* = 0.067) the ADFI on days 7 to 14. From days 7 to 20 (post-challenged period), increasing levels of MBB linearly increased (*P* < 0.05) G:F, with a quadratic effect (*P* < 0.05). When considering the days 7 to 28 period, compared to the NC, 0.0% MBB treatment reduced (*P* < 0.05) ADFI. In the overall period (days 0 to 28) 0.0% MBB treatment decreased (*P* < 0.05) ADFI. Increasing daily MBB intake (g/d), had a quadratic effect on the G:F at days 7 to 20 (maximum 0.89 at 0.71 g/d MBB intake; *P* < 0.05) and days 7 to 28 (maximum 0.65 at 0.75 g/d MBB intake; *P* < 0.05) of the study (Fig. 6A–B). The optimal level of MBB for G:F at days 7 to 20 was 0.22% and G:F at days 7 to 28 was 0.10% calculated in relation to the ADFI (622 and 322 g/d, respectively).

**Table 9.** Growth performance of nursery pigs fed diets supplemented with MBB^1^ under F18^+^*E. coli* challenge

	MBB				*P* value			
Item	NC^2^	0.0%	0.1%	0.2%	SEM^3^	NC vs. * *0.0%	Linear^4^	Quadratic^5^
Body weight, kg								
day 0	6.8	6.8	6.8	6.8	0.25	1.000	0.847	0.733
day 7	7.3	7.4	7.1	7.1	0.25	0.483	0.129	0.310
day 14	9.2	8.5	8.8	8.8	0.20	0.008	0.324	0.600
day 20	12.2	11.5	11.5	11.8	0.54	0.269	0.682	0.729
day 28	17.8	16.3	16.6	17.1	0.71	0.168	0.478	0.814
Average daily gain, g/d								
days 0 to 7 (Pre challenge)	68	84	40	45	17	0.496	0.845	0.730
days 7 to 14 (post-challenge)	273	183	215	216	24	0.011	0.323	0.602
days 7 to 20	382	331	334	358	33	0.269	0.680	0.730
days 7 to 28	502	435	446	471	32	0.167	0.476	0.814
days 0 to 28 (Overall)	391	341	348	366	25	0.179	0.538	0.748
Average daily feed intake, g/d								
days 0 to 7 (Pre challenge)	132	142	108	108	15	0.639	0.677	0.976
days 7 to14 (post-challenge)	314	256	264	310	21	0.054	0.067	0.451
days 7 to 20	534	484	389	444	50	0.336	0.391	0.146
days 7 to 28	770	632	589	644	41	0.026	0.844	0.395
days 0 to 28 (Overall)	610	505	474	512	32	0.024	0.901	0.394
Gain to feed ratio								
days 0 to 7 (Pre challenge)	0.45	0.45	0.20	0.25	0.11	0.977	0.841	0.487
days 7 to14 (post-challenge)	0.88	0.70	0.80	0.68	0.06	0.041	0.918	0.160
days 7 to 20	0.72	0.69	0.86	0.82	0.04	0.496	0.018	0.032
days 7 to 28	0.65	0.69	0.75	0.73	0.02	0.133	0.131	0.106
days 0 to 28 (Overall)	0.64	0.67	0.73	0.72	0.02	0.231	0.155	0.167

^1^MBB, microencapsulated blends of botanicals.

^2^NC, basal diet, without F18^+^*E. coli* challenge.

^3^SEM, standard error of means.

^4^Linear, linear effects of increasing levels of MBB under F18^+^*E. coli* challenge.

^5^Quadratic, quadratic effects of increasing levels of MBB under F18^+^*E. coli* challenge.

## Discussion

In the present study, direct oral challenge with F18^+^*E. coli* caused PWD, induced intestinal inflammation, increased immune response, and disrupted the integrity of the intestinal barrier, negatively impacting the growth performance of nursery pigs during the first week of challenge. These results were expected, as they align with previous studies conducted in the same facility using pigs of similar genetics and a similar research protocol (Duarte and Kim, 2022; Duarte et al., 2023b; Jang et al., 2023). The early weaning period, occurring at 21 d of age causes significant stress in pigs, disrupting the intestinal oxidative balance, leading to impaired intestinal barrier function (Cao et al., 2018), reduced nutrient absorption, and ultimately reduced growth performance (McLamb et al., 2013; Ming et al., 2021). Weaning stress also delays the development of the gastrointestinal and immune systems (Moeser et al., 2017; St-Pierre et al., 2023). The stress generated by weaning increases pigs’ susceptibility to intestinal infections such as F18^+^*E. coli*, which can colonize and proliferate in the intestinal tract (Cutler et al., 2007; Luppi, 2017). According to Duarte and Kim (2022), F18^+^*E. coli* has the potential to stimulate the excessive growth of pathogenic bacteria, negatively altering the balance of the jejunal mucosa-associated microbiota in nursery pigs. In this study, the pigs challenged with F18^+^*E. coli* and without any dietary treatment had an increase in the RA of *Staphylococcus saprophyticus* and *Bifidobacterium dentium* on day 7 post-challenge, potentially due to dysbiosis caused by the F18^+^*E. coli* infection. *Staphylococcus saprophyticus* is a bacterium that can have negative impacts by disseminating to distant organs and tissues, particularly following disruption of the intestinal barrier (Raz et al., 2005; Belizário and Faintuch, 2018). Interestingly, *Bifidobacteriaceae* is generally beneficial to the digestive tract (O’Callaghan and van Sinderen, 2016) and tended to increase during the F18^+^*E. coli* challenge in this study. This increase could suggest that *Bifidobacterium dentium* could grow under conditions where certain substrates become available, especially if pathogenic bacteria disrupt normal digestion or mucosal integrity in the jejunum (Engevik et al., 2021; Zhao et al., 2021).

In contrast, increasing levels of MBB showed the ability to modulate microflora composition, as evidenced by the reduction in the RA of *Staphylococcus saprophyticus*, *Staphylococcus kloosii*, *Staphylococcus saprophyticus-xylosus* on day 7, and *Prevotella copri* on day 21 post-challenge. Inclusion level of MBB at 0.1% could alter microbial dynamics, creating favorable conditions for the RA of *Helicobacter rappini,* which has been associated with both commensal and pathogenic infections depending on the host’s conditions (Schauer, 2001; Chaouche-Drider et al., 2009; Taillieu et al., 2022). However, the inclusion of 0.2% MBB reduced the RA of *Helicobacter rappini* on day 7 post-challenge. The ability of MBB to modulate microbiota could be primarily attributed to its bioactive compounds, such as terpenes and terpenoids, which disrupt bacterial membranes by interacting with their lipid components, altering permeability and causing leakage of intracellular contents (Di Matteo et al., 2024). Whereas these compounds affect both pathogenic and nonpathogenic bacteria, their impact varies by bacterial type and compound concentration (Trombetta et al., 2005; Valenzuela-Grijalva et al., 2017; Guimarães et al., 2019). Given the outer membrane composition of Gram-negative bacteria, including lipopolysaccharides, phospholipids, and lipoproteins (Beveridge, 1999), MBB bioactive compounds may have a stronger interaction with these bacteria (Połeć et al., 2022). The notable variations in the 0.1% MBB group, particularly in microbial diversity indices and specific taxa, may indicate sensitivity to MBB concentrations or host-microbiota interactions unique to this inclusion level. These variations could result from concentration-specific microbial responses to bioactive compounds in MBB, which can influence the stability of the intestinal microbiota. Future studies could benefit from exploring these dynamics further to elucidate the mechanisms behind such variability.

Furthermore, increasing levels of MBB increased the RA of beneficial bacteria such as *Lactobacillus mucosae* on day 7 post-challenge, known for its positive effects on feed efficiency and ileal morphological structure (Li et al., 2021b). However, a higher level (0.2%) of MBB modulated jejunal mucosa-associated microbiota by reducing the RA of *Lactobacillus salivarius* on day 21 post-challenge. This beneficial bacterium is known for its positive effects on intestinal functions, including anti-oxidative, immune-modulatory, and microbiota balancing (Yang et al., 2024). It is worth noting that the composition of the jejunal mucosa-associated microbiota differed between the 2 evaluation periods, implying that the microbiota population evolves as the nursery pigs grow and adapt to their diet. Additionally, the quadratic tendency observed in alpha diversity on day 7 post-challenge suggests that the inclusion of 0.1% MBB exerted selective pressure resulting in the lowest diversity among the MBB inclusion levels (Xia and Sun, 2023). This reduced diversity at 0.1% MBB may indicate a more targeted modulation of the microbiota, potentially enhancing the presence of beneficial microbes. Although a higher diversity, as seen with 0.2% MBB, is generally associated with greater resilience and stability (Hou et al., 2022), the selective effects of 0.1% MBB on microbiota composition could play a critical role in supporting recovery during the early stages of an F18^+^*E. coli* challenge.

The TLR signaling in the jejunum plays an essential role in the innate immune system, which is crucial for initiating immune responses against bacterial infections such as the F18^+^*E. coli* challenge (O’neill et al., 2013). In particular, lipopolysaccharide, a component found in the cell wall of Gram-negative bacteria such as F18^+^*E. coli*, is the primary stimulus for *TLR4* activation, one of the main TLR receptors. According to the results obtained in the present study, increasing levels of MBB tended to linearly reduce relative *TLR4* expression on day 7 and decrease it linearly on day 21 after the F18^+^*E. coli* challenge. This reduction could be related to the role of *TLR4* in regulating immune response, particularly in recognizing Gram-negative bacteria such as *E. coli*. Upon activation, *TLR4* triggers innate immune responses in macrophages and dendritic cells, contributing to the control of the enterotoxigenic infection spread (Vu et al., 2017). Notably, overactivation or unregulated stimulation of *TLR4* may result in detrimental inflammatory reactions, contributing to a range of pathological disorders and tissue damage, with the expenditure of a considerable amount of energy to sustain the inflammatory response and tissue healing in the intestine of pigs (Wang et al., 2013).

The *NOD* proteins represent a crucial family involved in recognizing pathogen-associated molecular patterns (Brubaker et al., 2015). In this study, F18^+^*E. coli* increases the *NOD2* expression on day 7 post-challenge. This increase could be attributed to the interaction between F18^+^*E. coli* and PAMP receptors. Similar to *TLR4*, NOD2 activation triggers downstream signaling pathways that activate nuclear factor-κB, leading to increased expression of pro-inflammatory cytokines in the intestine (Qin et al., 2018). The increase in pro-inflammatory cytokine secretion in nursery pigs challenged with F18^+^*E. coli* could reduce growth hormone levels and damage intestinal epithelial cells in the jejunum, compromising barrier function (Moeser et al., 2007; Arango-Duque and Descoteaux, 2014; Wang et al., 2019). In the present study, F18^+^*E. coli* producing STa and STb toxins stimulated IL-6 synthesis in the jejunum on day 7 post-challenge, primarily in the 0.0% MBB treatment, influenced by upstream signaling pathways (Loos et al., 2013; Wang et al., 2019). By day 21 post-challenge, the decrease in TNF-α, IL-6, and IL-8 levels in the jejunum of pigs challenged with F18^+^*E. coli* and 0.0% MBB indicates reductions in the pro-inflammatory response. These reductions were accompanied by an increase in IgG and IgA in the jejunum, suggesting activation of the humoral immune responses (Mantis et al., 2011; Duarte et al., 2023b). The responses could reflect a shift towards resolving intestinal inflammation and reducing tissue damage after the initial inflammatory activation (Duarte and Kim, 2022; Jang et al., 2023).

In contrast, a reduction in the inflammatory response was observed with increasing levels of MBB. On day 7 post-challenge, IL-6 contents in the jejunum were reduced, suggesting that phytobiotic compounds in MBB, such as terpenes and terpenoid molecules like thymol, could inhibit mRNA expression of IL-6 (Li et al., 2019). However, increasing levels of MBB elevated IL-8 levels in the jejunum on day 21 post-challenge, suggesting that MBB may induce a neutrophil response against the pathogen. Moreover, IL-8 signaling is also recognized for its mitogenic effects on epithelial cells (Zhu and Woll, 2005). In the context of intestinal recovery after challenge, IL-8 could promote the proliferation of epithelial cells, aiding in the healing and regeneration of the intestinal mucosa and potentially increasing cell proliferation after the F18^+^*E. coli* challenge (Zachrisson et al., 2001; Li et al., 2003; Laursen et al., 2014). Interestingly, daily MBB intake (g/d) had a quadratic effect on jejunal IgG contents, which may be related to the humoral immune response triggered by the F18^+^*E. coli* challenge (Mantis et al., 2011; Duarte et al., 2023b) and the bioactive compounds in MBB. In fact, terpenes and terpenoids in MBB may interact with B cells, inducing IgG production and stimulating the immune response (Nimmerjahn and Ravetch, 2010). The quadratic response indicates that higher concentrations of MBB potentially enhance immune signaling on day 21 post-challenge.

Intestinal tight junctions, a complex of proteins that connect adjacent enterocytes, are essential for preserving the integrity of the intestinal barrier and preventing the free passage of microorganisms across the paracellular space between cells (Bischoff et al., 2014). Key transmembrane proteins such as *OCLN* and *CLDN1*, along with cytosolic membrane-associated proteins like *ZO-1*, are crucial for the structural and functional integrity of these junctions (Shen et al., 2012; Zhou et al., 2018). The colonization of F18^+^*E. coli* generally has a detrimental effect on the intestinal barrier (Duarte et al., 2023a). As expected, the F18^+^*E. coli* infection decreased the relative gene expression of tight junction proteins (*OCLN* and *ZO-1*) on day 7 post-challenge, although a recovery effect was observed on day 21 post-challenge. The linear increase observed in the relative gene expression of tight junction proteins on 7 d post-challenge as MBB levels increased suggests that higher levels of MBB could support jejunal tight junction integrity in nursery pigs. However, on day 21 post-challenge, when the pathogenic infection was resolved, results indicate that the bioactive compounds of MBB may have an optimal level for maximizing their beneficial effects. This was demonstrated by the quadratic effect detected on the relative gene expression of *ZO-1*, where the high dose of MBB reduced *ZO-1* on day 21 post-challenge. When the negative effects of *E. coli* challenge resolved, the quadratic effect was observed, suggesting that under more stable conditions, MBB inclusion may help regulate *ZO-1* expression to a level that supports intestinal barrier function without overstimulation. The moderate dose of MBB (0.1%) produced the highest effect on *ZO-1* expression levels on day 21 post-challenge compared to the high dose (0.2%). Whereas higher *ZO-1* expression is generally associated with better tight junction integrity, the appropriate level may vary based on the recovery phase and physiological state of the jejunum. Notably, this effect was specific to *ZO-1*, as no changes were detected in related markers like *OCLN* and *CLDN1*. Further research is needed to understand the pathways through which MBB differentially influences barrier functions based on dose or the concurrent presence of intestinal health stressors.

The F18^+^*E. coli* challenge led to cellular damage and inflammatory response, which negatively affected cell proliferation in nursery pigs (Duarte et al., 2020; Xu et al., 2022), as observed in pigs challenged with 0.0% MBB, which had lower Ki-67^+^ cell numbers in the crypt compared to the NC on day 7 post-challenge. However, the inclusion of MBB tended to increase cell proliferation on day 7 post-challenge, indicating better preservation of the intestinal mucosa and an enhanced regenerative response (Chen et al., 2019; Gāliņa et al., 2020; Duarte and Kim, 2024) on day 7 post-challenge. This result supports the possible proliferative effect of IL-8 on the intestinal mucosa of pigs challenged with F18^+^*E. coli* and supplemented with MBB, as previously discussed. The VH was negatively affected by F18^+^*E. coli* on day 21 post-challenge, which is related to a reduction in nutrient absorption due to the smaller surface area of the damaged villi (Chwen et al., 2013). Interestingly, increasing levels and daily intake of MBB (g/d) showed a linear effect on VH:CD ratio on day 21 post-challenge, suggesting that high MBB inclusion has a beneficial effect on intestinal health and morphology. The improvement in intestinal morphology on day 21 in the presence of MBB may have been influenced by the higher Ki-67^+^ cell numbers observed on day 7 post-challenge. The increased proliferation rate of enterocytes after the F18^+^*E. coli* challenge might have contributed to faster renewal of the intestinal mucosa, potentially leading to quicker recovery and an improved intestinal structure later in the study.

Taken together, these results, including the reduction in the inflammatory response, enhancement of intestinal integrity, and increased presence of beneficial bacterial population, are supported by the linear tendency for decreased fecal score observed in the MBB treatments on day 10, and days 14 to 20 of the study. Thus, increasing the inclusion levels of MBB could improve overall intestinal health.

In the present study, no differences in growth performance were observed between 0.0%, 0.1%, and 0.2% MBB, except for a lower BW in 0.0% MBB on day 14 when compared to the NC, which was an expected result in this study. Nursery pigs were housed individually and fed for a short period, which may have limited the possibility of observing growth performance responses to F18^+^*E. coli.* However, the observed benefits on intestinal health likely contributed to the enhanced feed efficiency during the first 2 wk post-challenge (days 7 to 20) in pigs supplemented with increasing levels of MBB, suggesting improved nutrient utilization. Additionally, the quadratic effect observed with daily MBB intake on feed efficiency indicates that the combined benefits of improved intestinal health and reduced inflammation in challenged animals can maximize feed efficiency itself reaching a maximum, despite the F18^+^*E. coli* infection. A longer period might be necessary to fully capture growth performance responses in nursery pigs supplemented with MBB under challenge conditions.

In conclusion, the direct oral challenge with F18^+^*E. coli* negatively impacted the intestinal health of nursery pigs by inducing intestinal inflammation, increasing immune response, and disrupting the integrity of the intestinal barrier. However, increasing levels of MBB exhibited some modulatory effects on the jejunal mucosal-associated microbiota, with trends suggesting changes in alpha diversity and potential reductions in certain harmful bacteria. The MBB also provided protective effects on intestinal barrier function by enhancing tight junction proteins and supporting immune responses. Whereas the effects of MBB on intestinal microbiota were limited, the combined benefits observed in intestinal health, morphology, and feed efficiency highlight its potential as a dietary strategy to support nursery pigs. Future studies should further explore the mechanisms behind these effects and optimize inclusion levels. Increasing intake of MBB showed potential benefits for feed efficiency and intestinal health after F18^+^*E. coli* challenge at the range of 0.10% to 0.14%. Overall, the results suggest that MBB has the potential to mitigate the negative impacts of F18^+^*E. coli* challenge on the intestinal health and growth performance of nursery pigs. Increasing levels and intake of MBB could contribute to improving intestinal health, which could support feed efficiency in nursery pigs challenged with F18^+^*E. coli* by regulating the immune response, enhancing intestinal integrity function, and ameliorating intestinal morphology.

## Supplementary Material

skaf047_suppl_Supplementary_Material

## Abbreviations

ADFI: average daily feed intake
ADG: average daily gain
BW: body weight
*CLDN1*: claudin-1
E. coli: *Escherichia coli*
G:F: gain to feed ratio
IgA: immunoglobulin A
IgG: immunoglobulin G
IL-6: interleukin 6
IL-8: interleukin 8
MDA: malondialdehyde
*NOD1*: nucleotide-binding oligomerization domain containing 1
*NOD2*: nucleotide-binding oligomerization domain containing 2
*OCLN*: occludin
PWD: postweaning diarrhea
RA: , relative abundance
Sta: heat-stable toxins A
STb: heat-stable toxins B
*TLR2*: toll-like receptor 2
*TLR4*: toll-like receptor 4
TNF-α: tumor necrosis factor-alpha
VH: villus height
VH:CD: villus height to crypt ratio
*ZO-1*: zonula occludens-1
