# Supplementary material for: Investigation of the nutritional and functional roles of a microencapsulated blend of botanicals on intestinal health and growth of nursery pigs challenged with F18+Escherichia coli
Source: J Anim Sci. 2025 Feb 15;103:skaf047. doi: 10.1093/jas/skaf047 (PMC11956686; doi:10.1093/jas/skaf047)
Supplement: skaf047_suppl_Supplementary_Material [file skaf047_suppl_supplementary_material.docx]

Supplemental table 1. Sequence of primers for microbial sensing and intestinal integrity in the jejunum of nursery pigs fed with MBB^1^ under F18^+^ *E. coli* challenge.

| Gene^2^ | Primer sequences (5′–3′)^3^ | Accession number | Size |
| --- | --- | --- | --- |
| *GAPDH* | F: TCGGAGTGAACGGATTTGGC  R: TGCCGTGGGGTGGAATCATAC | NM_001206359.1 | 20 |
| *NOD1* | F: AACACCGATCCAGTGAGCAG  R: AAATGGTCTCGCCCTCCTTG | NM_001114277.1 | 230 |
| *NOD2* | F: GTGCCTCCCCTCTAGACTCA  R: ACGAACCAGGAAGCCAAGAG | NM_001105295.1 | 191 |
| *TLR2* | F: GGGCTGCGTTCATTCATCAG  R: CTGCAGAGGATGGATGGCAA | XM_005653576.3 | 132 |
| *TLR4* | F: CGTGCAGGTGGTTCCTAACA  R: GGTTTGTCTCAACGGCAACC | NM_001113039.2 | 326 |
| *CLDN1* | F: AAACCGTGTGGGAACAACCA  R: CACATGAAAATGGCTTCCCTC | NM_001244539.1 | 196 |
| *OCLN* | F: TCAGGTGCACCCTCCAGATT  R: AGGAGGTGGACTTTCAAGAGG | XP_005672579.1 | 169 |
| *ZO-1* | F: CAGAGACCAAGAGCCGTCC  R: TGCTTCAAGACATGGTTGGC | XM_003480423.4 | 105 |

^1^MBB, microencapsulated blends of botanicals.

^2^*NOD1, nod-like receptor 1; NOD2, nod-like receptor 2; TLR2, toll-like receptor 2; TLR4, toll-like receptor 4; CLDN1, claudin-1; OCLN, occludin; ZO-1, zonula occludens 1.*

^3^F, forward; R, reverse.

Supplemental table 2. Relative abundance of jejunal mucosa-associated microbiota at the phylum level in nursery pigs fed diets supplemented with MBB^1^ under F18^+^ *E. coli* challenge.

|  | MBB | | | | | | |  | | *P* value | | | | | |
| --- | --- | --- | --- | --- | --- | --- | --- | --- | --- | --- | --- | --- | --- | --- | --- |
| Item | NC^2^ | 0.0% | 0.1% | | 0.2% | | SEM^3^ | | NC *vs.* 0.0% | | Linear^4^ | | Quadratic^5^ | |  |
| D 7 post-challenge | |  | |  | |  | |  | |  | |  | |  | |
| Firmicutes | 47.7 | 58.9 | | 39.9 | | 50.6 | | 9.9 | | 0.391 | | 0.498 | | 0.170 | |
| Actinobacteria | 13.6 | 11.7 | | 13.3 | | 16.2 | | 3.8 | | 0.731 | | 0.361 | | 0.863 | |
| Proteobacteria | 32.1 | 20.0 | | 43.1 | | 28.3 | | 11.4 | | 0.450 | | 0.582 | | 0.146 | |
| Bacteroidetes | 5.4 | 5.3 | | 2.2 | | 3.6 | | 2.3 | | 0.965 | | 0.583 | | 0.389 | |
| Spirochaetae | <0.1 | 0.7 | | 0.1 | | 0.1 | | 0.3 | | 0.114 | | 0.198 | | 0.435 | |
| Cyanobacteria | 0.1 | 0.2 | | <0.1 | | 0.1 | | 0.1 | | 0.175 | | 0.243 | | 0.361 | |
| Lentisphaerae | <0.1 | 0.3 | | <0.1 | | 0.1 | | 0.1 | | 0.157 | | 0.301 | | 0.337 | |
| Others | 1.1 | 2.9 | | 1.4 | | 1.1 | | 1.0 | | 0.994 | | 0.206 | | 0.621 | |
| D 21 post-challenge | |  | |  | |  | |  | |  | |  | |  | |
| Firmicutes | 48.2 | 39.4 | | 56.0 | | 39.7 | | 12.4 | | 0.518 | | 0.976 | | 0.219 | |
| Actinobacteria | 5.5 | 15.0 | | 14.7 | | 11.2 | | 4.0 | | 0.093 | | 0.516 | | 0.759 | |
| Proteobacteria | 43.0 | 34.6 | | 15.9 | | 45.7 | | 13.1 | | 0.617 | | 0.453 | | 0.080 | |
| Bacteroidetes | 2.4 | 8.0 | | 10.0 | | 1.9 | | 2.7 | | 0.164 | | 0.126 | | 0.286 | |
| Cyanobacteria | 0.2 | 0.2 | | 0.7 | | 0.1 | | 0.2 | | 0.979 | | 0.806 | | 0.057 | |
| Others | 0.7 | 2.8 | | 2.7 | | 1.4 | | 0.5 | | 0.004 | | 0.072 | | 0.417 | |

^1^MBB, microencapsulated blends of botanicals.

^2^NC, basal diet, without F18^+^ *E. coli* challenge.

^3^SEM, standard error of means.

^4^Linear, linear effects of increasing levels of MBB under F18^+^ *E. coli* challenge.

^5^Quadratic, quadratic effects of increasing levels of MBB under F18^+^ *E. coli* challenge.
